# Elevated Serum ADA Activity as a Marker for Diagnosis and Prognosis of Visceral Leishmaniasis and Post Kala-Azar Dermal Leishmaniasis in Indian Patients

**DOI:** 10.1371/journal.pone.0154117

**Published:** 2016-05-17

**Authors:** Vijayamahantesh, Ajay Amit, Manas R. Dikhit, Raj K. Pandey, Kuljit Singh, Ritesh Mishra, V. N. R Das, Pradeep Das, Sanjiva Bimal

**Affiliations:** 1 Division of Immunology, Rajendra Memorial Research Institute of Medical Sciences, Patna, 800007, India; 2 Department of Biotechnology, National Institutes of Pharmaceutical Education and Research, Hajipur, 844102, India; 3 Department of Bioinformatics, Rajendra Memorial Research Institute of Medical Sciences, Patna, 800007, India; 4 Department of Clinical Medicine, Rajendra Memorial Research Institute of Medical Sciences, Patna, 800007, India; 5 Department of Molecular Biology, Rajendra Memorial Research Institute of Medical Sciences, Patna, 800007, India; 6 Department of Biochemistry, Rajendra Memorial Research Institute of Medical Sciences, Patna, 800007, India; Royal Tropical Institute, NETHERLANDS

## Abstract

Serum adenosine deaminase (ADA) activity increases in diseases where cellular immunity is involved. Since cell-mediated immune responses play a paramount role in the pathogenesis and healing of the visceral leishmaniasis, therefore, the present study was undertaken to evaluate the serum ADA activity in different pathological conditions. Adenosine deaminase was determined in sera of active visceral leishmaniasis (VL) patients (n = 39), active postkala-azar dermal leishmaniasis (PKDL) cases (n = 34) at the point of diagnosis and after treatment stages along with healthy controls (n = 30), endemic healthy subjects (n = 34) and endemic asymptomatic subjects (n = 34).Our *in-vitro* result revealed that monocytes secrete significant ADA level in response to *Leishmania donovani (L*.*donovani)* stimulation. The serum ADA activity in active VL and PKDL subjects were found to be significantly higher than that of respective treated cases and healthy controls. We also observed a marginal number (17.6%) of endemic asymptomatic subjects showed elevated serum ADA activity. Further, the ADA activity in PKDL was found to be decreased gradually during the different phases of treatment. Interestingly, 2 out of 32 treated VL cases found to have high serum ADA activity during follow up period were relapsed within few days. These results suggest the possibility of ADA as a marker of clinical pathogenesis and can be used as a surrogate marker in the diagnosis and prognosis of VL and PKDL.

## 1. Introduction

The leishmaniases are protozoan parasite disease caused by more than 20 *Leishmania* species that are transmitted to humans by the bites of infected sandflies. Among these, visceral leishmaniasis (VL) caused by *Leishmania donovani* (*L*.*donovani*), is the most severe in the Indian subcontinent and is fatal if untreated. Post kala-azar dermal leishmaniasis (PKDL) is a dermal complication, caused as a sequel to VL, it is characterised by a macular, maculopapular and nodular rash in a patient who has recovered from VL and who is otherwise well [1]. In India, it manifests in 5–15 per cent of VL cases after months or several years of remission from infection, while in Sudan, it develops within weeks or months in 50–60 per cent of cured VL cases [2,3]. Thus, it is prevalent in the areas where *Leishmania donovani* is the causative organism [1, 4].

Early and accurate diagnosis and treatment remain key factors to curb the VL. In addition, a proper prognostic marker fends off treatment failures. Microscopy remains the gold standard for the diagnosis of VL. Apart from this, rK39 based immuno-chromatographic test (ICT), direct agglutination test (DAT) and PCR have shown high diagnostic accuracy [5–7], but these have some inherent limitations. Although the specificity is high, the sensitivity of microscopy varies from organ to organ, spleen has highest sensitivity (93–99%) followed by bone marrow (53–86%) or lymph node (53–65%) aspirates [8, 9]. Apart from this, invasive method of sample collection needs the expertise and in some cases spleenic aspiration is fatal[10]. Antibody based immunostrip (rK39) are currently the best available diagnostic tool for VL for use in remote areas. rK39 based test showed sensitivity and specificity estimates of 93.9% and 95.3% respectively[5, 6]. However, this test has been shown to be less accurate in East Africa [11]. Moreover, a significant proportion of asymptomatic individuals test positive for the rK39 and the seroprevalence in healthy populations varies from <10% to up to >30% [12, 13], therefore, relapse can’t be diagnosed by this strip[14–16]. DAT has been extensively validated in most endemic areas, with sensitivity and specificity estimates of 94.8 and 97.1 respectively [12]. DAT may yield positive results for a long time after complete cure and thus has not proved to be of much prognostic value [17] and also it requires equipment such as microtitreplate and micropipettes. Although PCR based method gives higher sensitivity (93.80) [7], however, its sensitivity for the detection of *Leishmania* DNA in the blood of parasitologically proven VL cases was only 70% [18] and is poorly adapted to field settings [19]. In the absence of animal reservoir PKDL patients are thought to be the reservoirs for visceral leishmaniasis in Indian subcontinent and this necessitates an accurate diagnosis of PKDL[20]. Methods for the diagnosing PKDL mainly consists of clinical signs and symptoms and pre-history of VL, parasite demonstration on the skin, leishmanin skin test and immuno-chromatographic test [16]. The clinical signs may often confuse with other skin disorder [21]. Other methods often lack sensitivity or specificity as (i) only 20–40% positive parasite demonstration on the smear because of low parasite load [1] (ii) serological tests may be positive due to the past occurrence of VL (iii) the leishmanin skin test (LST) may or may not be positive. In addition, around 10% of cases have no history of VL, and 10% of cases have no positive serological test making a strict clinical definition important [21].

The assay of ADA activity in the serum and other biologic fluids is very useful for an accurate diagnosis of many pathological conditions [22, 23]. Although VL and PKDL are caused by the same parasite but these two diseases differ in many aspect of pathogenesis and disease progression due to their localization in the body [24]. Erel et al. found an increased lymphocytic specific ADA activity in patients with cutaneous leishmaniasis [25]. But reports on sera ADA levels in Indian VL are scarce, one study by Tripati et al showed increased ADA level in Indian VL patients during active VL and subsequent decreased ADA after treatment and another study by A.K.Rai et al was inconclusive as they didn’t not measure the serum ADA activity after treatment [26, 27]. Most importantly, since symptoms and pathophysiology of VL and PKDL differ considerably [28, 29], a study on serum ADA level between VL and PKDL will shed a new light on the diagnosis and better understanding of the pathophysiology of these diseases. Interestingly, a study from Nepal shows that the risk of seroconversion in asymptomatic was higher in Indian than in Nepalese [30]. Therefore, on this context, it is also important to access the serum ADA of asymptomatic subjects because diagnosis of asymptomatic leishmanial infection is hampered by the lack of validated definitions and appropriate biological markers. Here, our study is an attempt to investigate the serum ADA activities of patients with visceral leishmaniasis (VL), post kala-azar dermal leishmaniasis (PKDL) and endemic asymptomatic subjects in comparison with healthy control.

## 2. Materials and Methods

### 2.1 Clinical samples and diagnosis

#### Study groups of persons examined

VL, PKDL, asymptomatic and endemic and non-endemic healthy subjects of both sexes aged from 13 to 65 years were sampled from RMRI OPD, endemic area (District Saran, Bihar, India) and non endemic area (District Patna, Bihar, India). Fresh VL cases were treated with 10 mg/kg body weight single dose Ambisomeandfollowed up for three to six months after treatment. Confirmed PKDL patients were treated by infusion with amphotericin B deoxycholate (0.5 mg/kg body weight/day on days 1–20, 50–70 and 100–120) and followed up for three to six months after third course. The study was started after obtaining their informed and written consent. In case of minor, written consent was obtained from his/her guardian on his/her behalf. Approaching subjects unwilling to give their informed consent or were positive for tuberculosis, malaria, kidney, heart and liver diseases, HIV or with symptoms of asthma or rheumatic arthritis were excluded from this study. Diagnosis was confirmed by demonstration of amastigotes in spleenic/bone marrow aspirate or microscopically. Asymptomatic cases where defined as endemic area individuals with rK39 positive without signs and symptoms.

#### Group 1

Thirty nine (39) newly diagnosed patients with visceral leishmaniasis. All the active VL patients presented characteristic signs and symptoms of the disease and diagnosis was confirmed by the presence of *L*.*donovani* either in Giemsa stained bone marrow aspirate or spleenic aspirate.

#### Group 2

Thirty (30) successfully treated VL cases (Treated with single dose Ambisome) without thepathospleenomegaly and feverfrom group1 were included.

#### Group 3

Thirty four (34) clinically diagnosed PKDL patients of different group (macular/popular lesions) were included in this study.

#### Group 4

Twenty nine (29) treated PKDL patients (three courses, every course consists of Amphotericin B deoxycholate 0.5 mg/kg body weight/day for twenty days) with decreased or no macular or popular lesions were considered.

#### Group 5

Thirty four (34) endemic asymptomatic persons, with positive rK39, apparently without signs and symptoms of the diseases were included.

#### Group 6

Thirty (30) non-endemic healthy subjects, with rK39 negative and without sign and symptoms of the disease and no case history of leishmaniasis.

#### Group 7

Thirty four (34) endemic healthy subjects with rK39 negative and without any signs and symptoms of the diseases were considered for further study.

### 2.2 Serum collection

Blood samples were collected in plane blood collection vials (Becton Dickinson, San Diego,USA) and let it for room temperature for 30 min and upper serum is transferred to sterile eppendorf tube and centrifuged at 3000 rpm for 10 min at 4°C. 20 μl serum supernatant was used for measurement of ADA activity and rest is stored at -20°Cfor future usage.Blood collected in thefield were transported in ice packs and serum was separated and ADA assay was performed immediately or stored at -20°C and used for ADA assay within a week. For PKDL, blood sample was collected at the start of the treatment and ADA assay was carried out on the same day and rest is stored at -20°C for future usage.

### 2.3 Preparation of cell lysate

Mononuclearcytes and granulocytes were separated by buffy coat method [31]. Cells were resuspended in sterile distilled water and lysed by sonication. Lysate was centrifuged at 8000 rpm for 10 min at 4°C and 50 μg of supernatant protein was used for measurement of ADA activity and rest is stored at -20°Cfor further usage.

### 2.4 ADA activity in monocytes culture supernatant

PBMCs were isolated from five healthy donor and 2 x 10^6^ cells per well seeded on six well plate and incubated overnight at 37^0^ C CO_2_ incubator. Non-adherent cells were washed out and monocytes were pulsed with 1:10 monocytes to metacyclic *Leishmania donovani* promastigotes for 12–16 hrs. Culture supernatant was collected after 48 hours and used for measurement of ADA activity.

### 2.5 Measurement of ADA activity

ADA activity was measured by ADA-MTB kit (Tulip Diagnostic, India) according to the manufactured protocol.

### 2.6 Statistical analysis

Student’s two-tailed paired t test or unpaired t test was used to determine the difference between the groups studied. Statistical analysis was performed using GraphPad Prism 5, USA software and all the data are expressed as Mean ± SEM (standard error of the mean). P<0.05 was considered statistically significant for all the analysis.

## 3. Results

### 3.1 Mononuclear cells are the source of serum ADA level during *Leishmania donovani* infection

**Fig 1:**
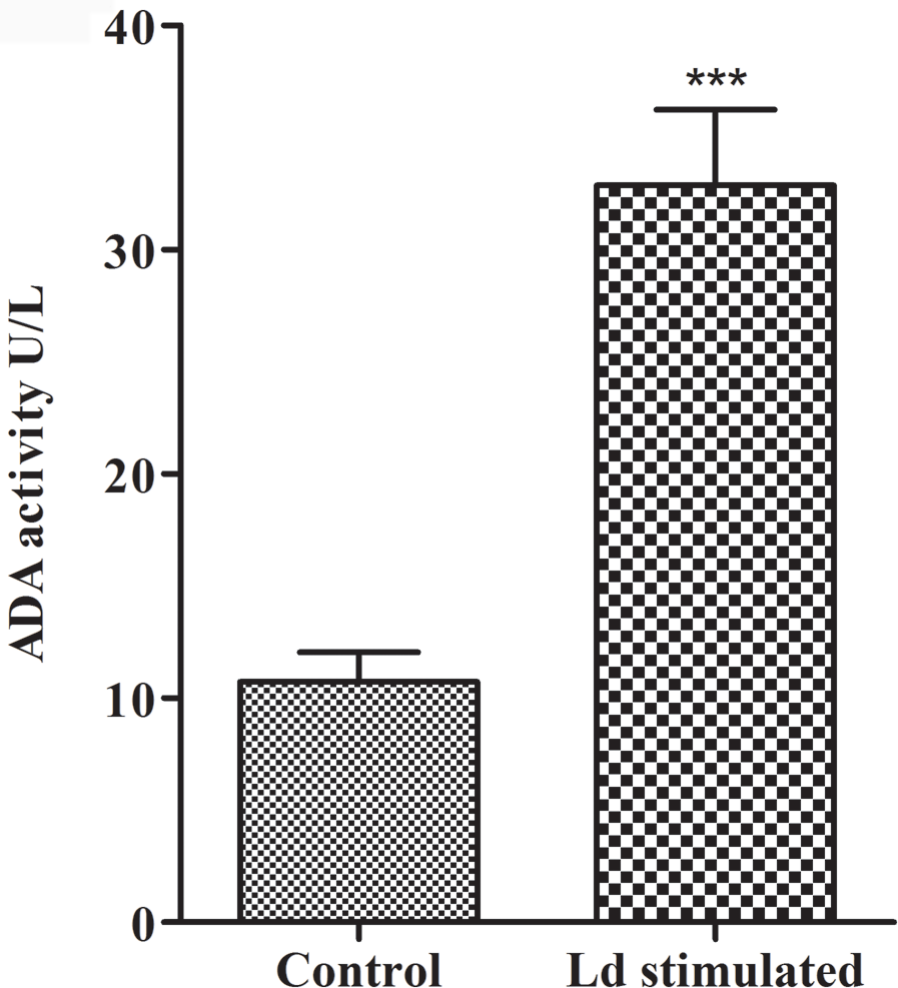
ADA activity in culture supernatant: PBMCs (n = 10) were cultured on 6 well plate and adherent monocytes were stimulated with 1:10 metacyclic *L*.*donovani*. After 48 hrs culture supernatant was collected and used for ADA activity. *** = Level of significance p<0.001. n = number of subjects.

**Fig 2:**
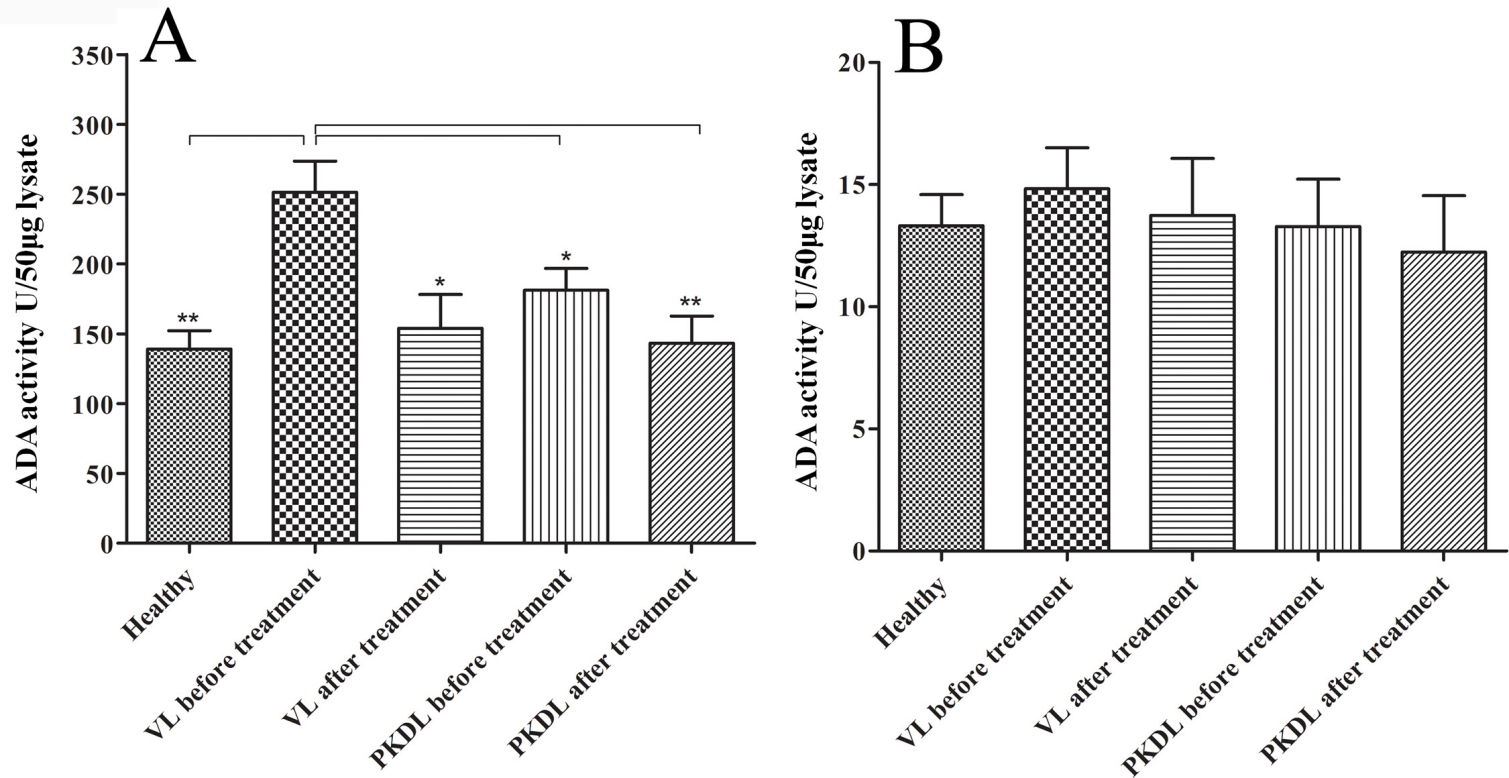
Intracellular ADA activity: mononuclear cells and granulocytes were isolated from whole blood. Intracellular ADA activity of mononuclear cells (A) and granulocyte lysate (B) was measured in visceral leishmaniasis and post kala-azar dermal leishmaniasis before and after treatment along with healthy subjects. * = Level of Significance p<0.05. ** = Level of Significance p<0.01. N = 10.

*In vitro* stimulation of monocytes with *L*.*donovani* revealed a significant increased ADA activity in culture supernatant as compared to unstimulated/control monocytes (Fig 1, p<0.001). Further, we isolated the mononucleocytes and granulocytes from whole bloods of VL and PKDL in pre and post treatment stage and evaluated the intracellular ADA levels from mononuclear cells and granulocytes from all the subjects. We found significantly higher ADA level in mononuclear cell lysate in VL pre-treatment stages compared to VL post treatment stages and healthy control (Fig 2A, p<0.05), whereas in PKDL cases although active PKDL cases shows high ADA activity compared to treated PKDL cases but difference was not statistically significant (Fig 2A, p>0.05). Further, active VL cases showed significantly higher ADA activity compared to active PKDL, treated PKDL and healthy control (Fig 2A, p<0.05).We could not find any significant difference in ADA activity of granulocytes lysate between these groups (Fig 2B, p>0.05).

### 3.2 Serum ADA activity is elevated during active VL and PKDL disease

**Fig 3:**
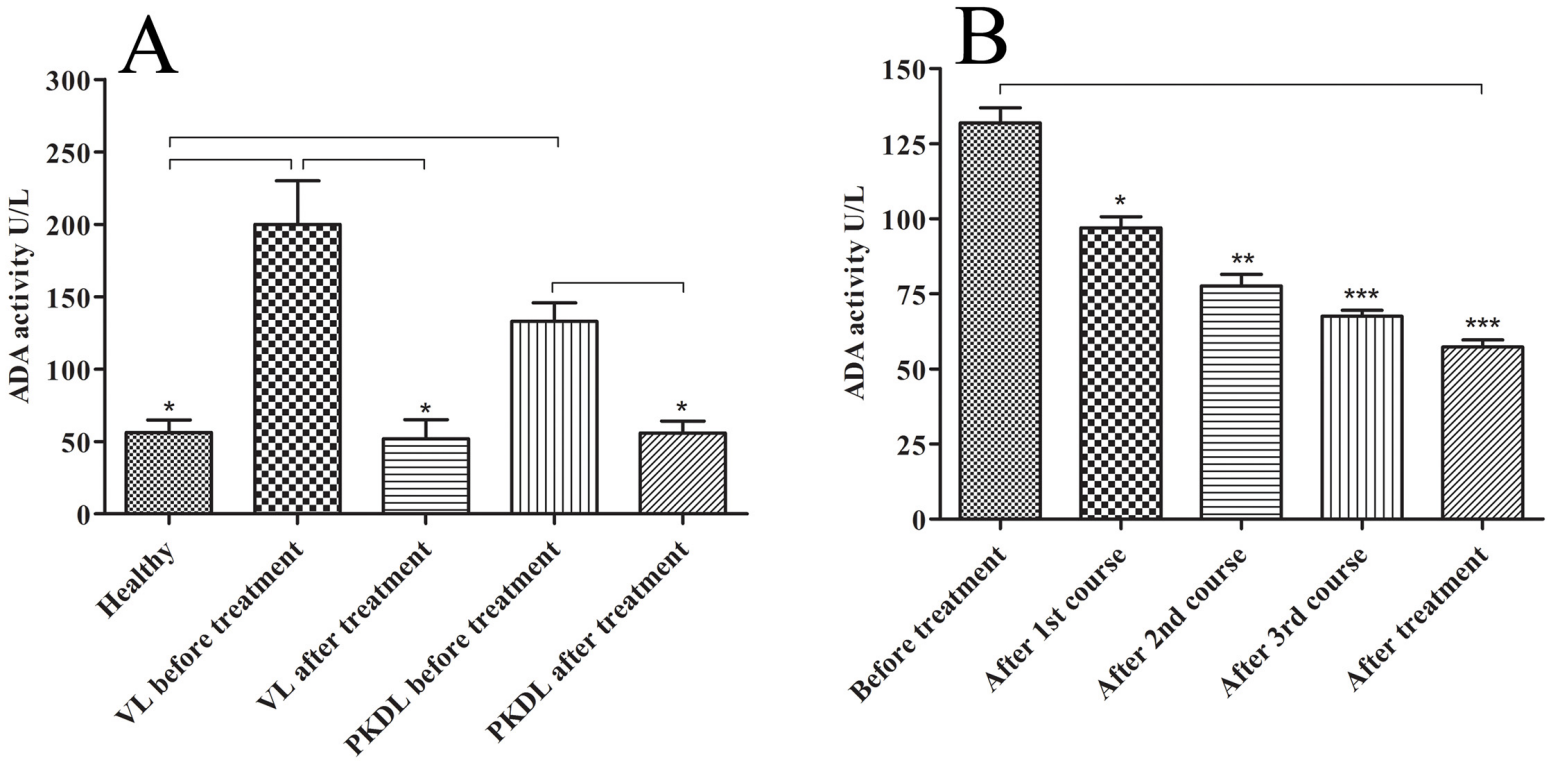
Serum ADA activity of active VL (n = 39), PKDL (n = 34) and healthy subjects (n = 30) and post treatment stages of VL (n = 30) and PKDL (n = 29) (**A**). Serum ADA activity of PKDL, at the point of diagnosis (n = 30), on day 20 (n = 25), on day 70 (n = 22), on day 120. (n = 19) and after complete treatment (n = 29) (**B**).

The experiment was designed to evaluate the level of serum ADA activity in active VL and PKDL patients in their pre and post treatment. A significantly increased serum ADA activity was found in active VL and PKDL pre-treatment stages compared to their respective post treatment stages and healthy subjects (Fig 3A, p<0.05). Such higher ADA activity in serum was, however, reduced 3.08 and 2.30 fold following successful treatment of VL and PKDL subject respectively (Fig 3A, p <0.05). However, we observed a significantly higher serum activity in active VL cases compared to active PKDL cases (Fig 3A, p<0.05). Further, the serum ADA activity in PKDL was found to be decreased gradually as the treatment progresses (Fig 3B, p<0.05).

### 3.3 Asymptomatic subjects shows uneven ADA activity

**Fig 4:**
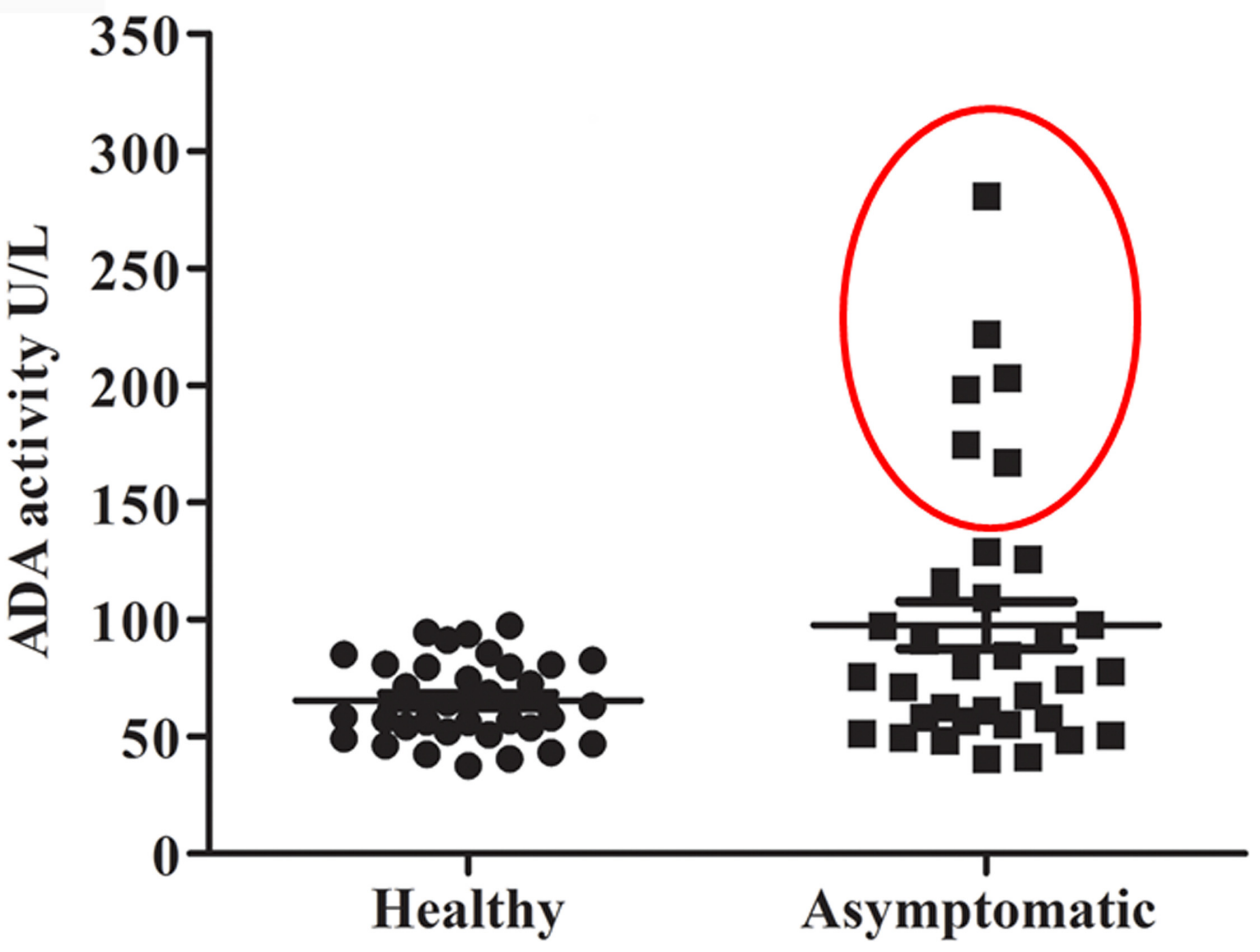
Serum ADA activity of endemic healthy subjects (n = 34) and endemic asymptomatic (n = 34) subjects. 17.6% (6 out of 34) asymptomatic subjects possessed higher ADA activity (marked with red circle).

Since, a marginal number of asymptomatic individuals will convert into VL clinical manifestations [32]. To known the status of serum ADA activity of asymptomatic individuals, we measured the serum ADA activity of endemic asymptomatic subjects and endemic healthy subjects from endemic area. The serum ADA level of endemic healthy subjects found to be in normal range, but the asymptomatic subjects showed a variation (Fig 4), 17.6% (six out of thirty four) asymptomatic subjects found to possess elevated serum ADA level.

## 4. Discussion

The ADA activity originates from the action of two principal isoenzymes, ADA-1 and ADA-2, which have different pH, Michaelis constant (Km) and relative substrate specificity patterns [33]. ADA-1 is present in all tissues and is essential for an efficient immune response whereas ADA-2 is exclusively present in monocytes and macrophages and is secreted in response to pathogen [34, 35]. It has been suggested that elevated serum ADA activity reflects the proliferation of T lymphocytes and monocytes/macrophage activity to microorganism or their antigen [36, 37]. However, depleted T lymphocytes are associated with leishmaniasis [38, 39]. So, we presume that in lymphocyte depleted condition, the elevated serum ADA might be contributed by monocytes during visceral leishmaniasis. To test our hypothesis, we stimulated monocytes with *Leishmania donovani in vitro* and measured the secreted ADA level in culture supernatant. We found a significantly higher ADA activity in *leishmania* stimulated culture supernatant compared to unstimulated (Fig 1, p<0.001, S1 Table). Hence, this result suggests that monocytes also contribute to the secretion of ADA during *Leishmania donovani* infection. However, during *leishmania* infection, neutrophils are the first cells to reach the site of infection [40]. Therefore, to test whether granulocytes contribute to the production of ADA or not, we measured the intracellular ADA activity in mononuclear cells and granulocyte lysate. We found a significantly increased ADA activity of mononuclear cell lysate in active VL, compared to active PKDL, treated VL and PKDL and healthy subjects (Fig 2A, p<0.05, S2 Table). However, we could not find any difference in ADA activity of mononuclear cell lysate between active and treated PKDL (Fig 2A, P>0.05, S2 Table). This is attributed to the difference in the systematic and local immune response. In previous study also it has been observed that the immune response of PKDL peripheral blood mononuclear cells varies from that of nodular/macular cells [24]. Interestingly, we could not find any significant difference in granulocyte lysate ADA activity between VL, PKDL and healthy control before or after treatment (Fig 1, p>0.05). Our intracellular granulocyte ADA activity results are in agreement with findings of Erel et al [25]. So, these findings indicate that mononuclear cells contribute to the elevated serum ADA activity during infectious diseases.

Elevated serum ADA activities have been observed in many infectious diseases mainly involving the macrophages [22, 23, 41, 42] and it has been considered as a marker of cell mediated immunity [43, 44]. In our subsequent experiments, we measured the serum ADA level in VL and PKDL patients in their pre and post treatment stages. The level of serum ADA was found significantly higher in Indian patients with VL and PKDL at pre-treatment stage compared to healthy subjects (Fig 3A, p<0.05, S3 Table). After successful treatment the serum ADA level decreased to 3.0 and 2.3 fold in VL and PKDL patients respectively (Fig 3A, p<0.05) and could not find any significant difference between VL and PKDL after treatment (Fig 3A, p<0.05, S3 Table). Interestingly, we found a significantly increased ADA level in active VL compared to active PKDL patients (Fig 3A, p<0.05). This discrepancy may be due to difference in immuno-pathogenesis of both forms of disease, VL is more aggressive and systemic disease, whereas PKDL is comparatively mild and restricted to the skin lesions[28, 45, 46]. Further, a linear correlation was observed between treatment course and serum ADA level in PKDL patients, serum ADA level subsides as the course of the treatment proceeds (Fig 3B, p<0.05, S3 Table).Due to single dose Ambisome drug regime, the ADA activity could not be measured during mid treatment in VL cases. Interestingly, 2 out of 32VL subjects (K-442/14 and K-483/14) showed elevated serum ADA activity during follow up and within a week, they are admitted as relapse cases. These results indicate the correlation between the serum ADA level and onset of the disease, which emphasises its prognostic value.

Asymptomatic individuals have been shown to harbour the parasite in their blood [47] and these cases can act as potential reservoirs in transmission dynamics of leishmaniasis. Multiple lines of evidences revealed that, a different degree of asymptomatic individuals (5%-30%) convert to VL clinical manifestation [47–48]. The intriguing thing, why particular asymptomatic individuals will develop VL clinical manifestation and rest will self cured has not been solved yet [49]. Relapse cases in our study gave a clue that elevated serum ADA might be a cue for the onset of the disease. Therefore, we presumed that all the asymptomatic might not possesses similar serum ADA activity. To test this hypothesis, we evaluated the serum ADA activity of endemic healthy subjects and rK39 positive asymptomatic subjects. The serum ADA activity of endemic healthy subjects found to be par with normal range, but, asymptomatic subjects exhibit uneven serum ADA activities. Only 17.6% (six out of thirty four) of the endemic asymptomatic subjects showed elevated serum ADA activities (Fig 4, S4 Table). This result notions the plausibility that, these individuals may harbour the parasite in their body. Interestingly, previous reports suggest that, the immune response of asymptomatic individuals to leishmanial antigen is similar to cured VL patients [24]. Although, asymptomatic individuals and cured VL patients shown the similar immune response but there was a great variability in the response of asymptomatic subject to leishmanial antigen [50]. This variation might be attributed to the plausibility that majority of the asymptomatic individuals might behave like cured patients and rest might behave like VL patients. Similarly, in our study marginal number (17.6%) of the asymptomatic individuals showed elevated serum ADA activity which is similar to active VL or PKDL cases and rest showed normal serum ADA activity which is similar to healthy or treated patients (Fig 4).This evidence points to the conjecture that marginal number of the asymptomatic subjects may show the cell mediated immune response which results in the elevated ADA level in the serum. In summary, our analysis shows that, mononuclear cells secretes ADA in response to leishmanial infection and the elevated serum ADA activity is associated with VL and PKDL, and serum ADA activity can be used as prognostic marker to monitor the course of the treatment.

## Conclusion

Visceral leishmaniasis (VL) constitutes a serious public health problem in endemic regions. Failure of early diagnosis jeopardizes the patient of an unfavourable evolution of the disease. In this context, an accurate diagnostic and an unambiguous prognostic marker will be a valuable tool for effective clinical practice. But, existing methods of leishmaniasis diagnosis and prognosis are hampered by the inherent limitations. On the other hand, ADA assay could be used as an alternative method of screening individuals with suspected VL and PKDL cases or as a tool for monitoring the efficacy of treatment and the appearance of relapse of subclinical disease. In our opinion, it is prudent to use rK39 strip and ADA in tandem, because seropositivty for rK39 and elevated serum ADA always associated with clinical leishmaniasis. Most importantly, this test is easy to perform, doesn’t required expertise and cold storage, therefore this test is well suited for field condition. However, we feel a longitudinal study on this is needed for further evaluation of this test.

## Supporting Information

S1 Table ADA (U/L) activity in culture supernatant. (XLSX)

S2 Table Intracellular ADA Activity U/50 μg lysate between Mononuclear and granulocytes. (XLSX)

S3 Table Serum ADA activity (U/L) in Healthy, VL and PKDL in pre and post treatment stages. (XLSX)

S4 Table Serum ADA activity (U/L) in Endemic healthy and Asymptomatic subjects. (XLSX)

## References

[1] Zijlstra EE, Musa AM, Khalil EAG, El Hassan IM, El-Hassan A. M.. Post-kala-azar dermal leishmaniasis. Lancet Infect Dis. 2003; 3(2): 87–98. 12560194 10.1016/s1473-3099(03)00517-6

[2] Ramesh V, Mukherjee A. POST‐KALA‐AZAR DERMAL LEISHMANIASIS. Int JDermatol. 1995; 34(2): 85–91. 7737782 10.1111/j.1365-4362.1995.tb03584.x

[3] Zijlstra EE, El-Hassan AM. Leishmaniasis in Sudan. 4. Post kala-azar dermal leishmaniasis. Trans R Soc Trop Med Hyg. 2001; 95(Supplement 1): S59–S76. 11370251 10.1016/s0035-9203(01)90219-6

[4] mahantesh Vijaya, Amit A, Kumar S, Dikhit MR, Jha PK, Singh AK, et al Up regulation of A2B adenosine receptor on monocytes are crucially required for immune pathogenicity in Indian patients exposed to Leishmania donovani. Cytokine. 2016; 79:38–44. 10.1016/j.cyto.2015.12.016 26748211

[5] Sundar S, Reed SG, Singh VP, Kumar PC, Murray HW. Rapid accurate field diagnosis of Indian visceral leishmaniasis. The Lancet. 1998; 351(9102): 563–565. 10.1016/S0140-6736(97)04350-X 9492776

[6] Chappuis F, Rijal S, Soto A, Menten J, Boelaert M. A meta-analysis of the diagnostic performance of the direct agglutination test and rK39 dipstick for visceral leishmaniasis. Br Med J.2006; 333(7571): 723. 16882683 10.1136/bmj.38917.503056.7C PMC1592383

[7] Piarroux R, Gambarelli F, Dumon H, Fontes M, Dunan S, Mary C, Quilici M. Comparison of PCR with direct examination of bone marrow aspiration, myeloculture, and serology for diagnosis of visceral Leishmaniasis in immunocompromised patients. J Clin Microbiol. 1994; 32(3): 746–749. 8195388 10.1128/jcm.32.3.746-749.1994 PMC263118

[8] Zijlstra EE, Ali M S, El-Hassan AM, El-Toum IA, Satti M, Ghalib HW. Kala-azar: a comparative study of parasitological methods and the direct agglutination test in diagnosis.Trans R Soc Trop Med Hyg.1992; 86(5): 505–507. 1475815 10.1016/0035-9203(92)90086-r

[9] Ho EA, Soong TH, Li Y. Comparative merits of sternum, spleen and liver punctures in the study of human visceral leishmaniasis.Trans R Soc Trop Med Hyg. 1948; 41(5): 629–636. 18912189 10.1016/s0035-9203(48)90458-1

[10] Kager PA, Rees PH. Splenic aspiration. Review of the literature. Trop Geog Med. 1983;35(2): 111–124. 6351381

[11] Boelaert M, El-Safi S, Hailu A, Mukhtar M, Rijal S, Sundar S et al Diagnostic tests for kala-azar: a multi-centre study of the freeze-dried DAT, rK39 strip test and KAtex in East Africa and the Indian subcontinent.Trans R Soc Trop Med Hyg. 2008; 102(1): 32–40. 17942129 10.1016/j.trstmh.2007.09.003

[12] Chappuis F, Sundar S, Hailu A, Ghalib H, Rijal S, Peeling RW.Visceral leishmaniasis: what are the needs for diagnosis, treatment and control?. Nat Rev Microbiol.2007; 5(11): 873–882. 17938629 10.1038/nrmicro1748

[13] Sinha PK, Bimal S, Pandey K, Singh SK, Ranjan A, Kumar N. A community-based, comparative evaluation of direct agglutination and rK39 strip tests in the early detection of subclinical Leishmania donovani infection. Ann Trop MedParasitol. 2008; 102(2): 119–125. 10.1179/136485908X252278 18318933

[14] Silva LDA, Romero HD, Prata A, Costa RT, Nascimento E, Carvalho SFG.Immunologic tests in patients after clinical cure of visceral leishmaniasis. Am JTrop Med Hyg.2006; 75(4): 739–743. 17038704

[15] Hailu A. Pre-and post-treatment antibody levels in visceral leishmaniasis.Trans R Soc Trop Med Hyg. 1990; 84(5): 673–675. 2278067 10.1016/0035-9203(90)90141-z

[16] Adams ER, Versteeg I, Leeflang MM. Systematic review into diagnostics for post-kala-azar dermal leishmaniasis (PKDL). J Trop Med. 2013. 10.1155/2013/150746 PMC3723149 23935641

[17] El Harith A, Kolk AH, Leeuwenburg J, Muigai R, Huigen E, Jelsma T et al Improvement of a direct agglutination test for field studies of visceral leishmaniasis. JClin Microbiol. 1988; 26(7): 1321–1325. 3410946 10.1128/jcm.26.7.1321-1325.1988 PMC266601

[18] Osman OF, Oskam L, Zijlstra EE, Kroon NC, Schoone GJ, Khalil ET etal Evaluation of PCR for diagnosis of visceral leishmaniasis.JClin Microbiol. 1997; 35(10): 2454–2457. 9316888 10.1128/jcm.35.10.2454-2457.1997 PMC229991

[19] Sreenivas G, Ansari NA, Singh R, Raju BS. Diagnosis of visceral leishmaniasis: comparative potential of amastigote antigen, recombinant antigen and PCR. Br JBiomed Sci. 2002; 59(4): 218. 12572956

[20] Thakur CP, Kumar K. Post kala-azar dermal leishmaniasis: a neglected aspect of kala-azar control programmes. Ann TropMedParasitol. 1992; 86(4): 355–359. 10.1080/00034983.1992.11812678 1463355

[21] Salotra P, Singh R. Challenges in the diagnosis of post kala-azar dermal leishmaniasis. Indian J Med Res. 2006; 123(3): 295 16778312

[22] Tsuboi I, Sagawa K, Shichijo S, Yokoyama MM, Ou DW, Wiederhold MD. Adenosine deaminase isoenzyme levels in patients with human T-cell lymphotropic virus type 1 and human immunodeficiency virus type 1 infections. Clin Diag Lab Immunol. 1995; 2(5): 626–630. 10.1128/cdli.2.5.626-630.1995 PMC170210 8548545

[23] Ri G, Ohno S, Furutani M, Furutani Y, Tsukahara T, Hagita N. An indication for correlation between the serum ADA level and gastric cancer risk. Anticancer Res.2010; 30(6): 2347–2349. 20651391

[24] Ismail A, El Hassan AM, Kemp K, Gasim S, Kadaru AEGM, Møller T, Theander TG. Immunopathology of post kala‐azar dermal leishmaniasis (PKDL): T‐cell phenotypes and cytokine profile. J Pathol. 1999; 189(4): 615–622. 10629566 10.1002/(SICI)1096-9896(199912)189:4<615::AID-PATH466>3.0.CO;2-Z

[25] Erel O, Kocyigit A, Gurel MS, Bulut V, Seyrek A, Ozdemir Y. Adenosine deaminase activities in sera, lymphocytes and granulocytes in patients with cutaneous leishmaniasis. Mem Inst Oswaldo Cruz.1998; 93(4): 491–494. 9711339 10.1590/s0074-02761998000400014

[26] Tripathi K, Kumar R, Bharti K, Kumar P, Shrivastav R, Sundar S, Pai K. Adenosine deaminase activity in sera of patients with visceral leishmaniasis in India. Clin Chim Acta. 2008; 388(1): 135–138. 18023276 10.1016/j.cca.2007.10.022

[27] Rai AK, Thakur CP, Velpandian T, Sharma SK, Ghosh B, Mitra DK. High concentration of adenosine in human visceral leishmaniasis despite increased ADA and decreased CD73. Parasite immunology.2011; 33(11): 632–636 10.1111/j.1365-3024.2011.01315.x 21729107

[28] Gasim S, Elhassan AM, Khalil EAG, Ismail A, Kadaru AMY, Kharazmi A. High levels of plasma IL-10 and expression of IL-10 by keratinocytes during viscera leishmaniasis predict subsequent development of post-kala-azar dermal leishmaniasis. Clin Exp Immunol.1998; 111: 64–69. 9472662 10.1046/j.1365-2249.1998.00468.x PMC1904865

[29] Gilad J, Borer A, Hallel-Halevy D, Riesenberg K, Alkan M, Schlaeffer F. Post-kala-azar dermal leishmaniasis manifesting after initiation of highly active anti-retroviral therapy in a patient with human immunodeficiency virus infection. Isr Med Assoc J: IMAJ.2001; 3(6): 451 11433642

[30] Picado A, Ostyn B, Singh SP, Uranw S, Hasker E, Rijal S. Risk factors for visceral leishmaniasis and asymptomatic Leishmania donovani infection in India and Nepal. PloS one. 2014;9(1): e87641 10.1371/journal.pone.0087641 24498159 PMC3909193

[31] Böyum A. A one-stage procedure for isolation of granulocytes and lymphocytes from human blood. General sedimentation properties of white blood cells in a 1g gravity field. Scand J Clin Lab Inv. Supplementum.1968; 97: 51. 4179067

[32] Sharma MC, Gupta AK, Das VNR, Verma N, Kumar N, Saran R et al Leishmania donovani in blood smears of asymptomatic persons. Acta tropica.2000; 76(2): 195–196. 10936579 10.1016/s0001-706x(00)00068-1

[33] Gakis C, Cappio‐Borlino A, Pulina G. Enzymes (Isoenzyme System) as homeostatic mechanisms the isoenzyme (ADA2) of adenosine deaminase of human monocytes‐macrophages as a regulator of the 2′ deoxyadenosine. IUBMB Life. 1998; 46(3):487–494. 10.1080/15216549800204012 9818088

[34] Conlon BA, Law WR. Macrophages are a source of extracellular adenosine deaminase‐2 during inflammatory responses. Clin Exp Immunol.2004; 138(1): 14–20. 15373900 10.1111/j.1365-2249.2004.02591.x PMC1809181

[35] Zuckerman SH, Olson JM, Douglas SD. Adenosine deaminase activity during in vitro culture of human peripheral blood monocytes and pulmonary alveolar macrophages. Exp Cell Res.1980; 129(2): 281–287. 7428821 10.1016/0014-4827(80)90494-2

[36] Gakis C, Calia G, Naitana A, Pirino D, Serru G. Serum adenosine deaminase activity in HIV positive subjects. A hypothesis on the significance of ADA2. Panminerva medica.1988 31(3), 107–113. 2689968

[37] Ungerer JP, Oosthuizen HM, Bissbort SH, Vermaak WJ. Serum adenosine deaminase: isoenzymes and diagnostic application. ClinChem. 1992;38(7): 1322–1326. 1623598

[38] Hailu A, van Baarle D, Knol GJ, Berhe N, Miedema F, Kager PA et al T cell subset and cytokine profiles in human visceral leishmaniasis during active and asymptomatic or sub-clinical infection with Leishmania donovani. Clin Immunol.2005; 117(2):182–191. 16125466 10.1016/j.clim.2005.06.015

[39] Cenini P, Berhe N, Hailu A, McGinnes K, Frommel D. Mononuclear cell subpopulations and cytokine levels in human visceral leishmaniasis before and after chemotherapy. J Infect Dis.1993; 168(4): 986–993. 8376845 10.1093/infdis/168.4.986

[40] Sousa LMA, Carneiro MBH, Resende ME, Martins LS, Dos Santos LM, Vaz LG.Neutrophils have a protective role during early stages of Leishmania amazonensis infection in BALB/c mice. Parasite Immunol.2014; 36(1): 13–31. 10.1111/pim.12078 24102495 PMC4307027

[41] Chaudhary SD, Gupta V, Saini AS, Singh V, Lal H. Adenosine deaminase activity in leprosy (a preliminary study). Indian J Leprosy.1988; 60(1): 17–20. 3204272

[42] Dikensoy O, Namiduru M, Hocaoglu S, Ikidag B, Filiz A. Increased pleural fluid adenosine deaminase in brucellosis is difficult to differentiate from tuberculosis. Respiration.2002; 69(6): 556–559. 12457012 10.1159/000066465

[43] Piras M, Gakis C, Budroni M, Andreoni G. Adenosine deaminase activity in pleural effusions: an aid to differential diagnosis. BrMedJ. 1978; 2(6154): 1751. 10.1136/bmj.2.6154.1751-a PMC1610017 737480

[44] Baganha MF, Âªgo AP, Lima MA, Gaspar EV, Cordeiro AR. Serum and pleural adenosine deaminase. Correlation with lymphocytic populations. CHEST J.1990; 97(3): 605–610. 10.1378/chest.97.3.605 1689629

[45] Van Der Poll T, Zijlstra EE, Mevissen M. Interleukin 6 during active visceral leishmaniasis and after treatment. ClinImmunolImmunopathol.1995; 77(1): 111–114. 10.1016/0090-1229(95)90144-2 7554475

[46] Ansari NA, Saluja S, Salotra P. Elevated levels of interferon-γ, interleukin-10, and interleukin-6 during active disease in Indian kala azar. Clin Immunol.2006; 119(3): 339–345. 16540374 10.1016/j.clim.2006.01.017

[47] Goswami RP, Bairagi B, Kundu PK. K39 strip test-easy, reliable and cost-effective field diagnosis for visceral leishmaniasis in India. JAssocPhysicians India. 2003; 51: 759–761. 14651134

[48] Topno RK, Das VN, Ranjan A, Pandey K, Singh D, Kumar N. Asymptomatic infection with visceral leishmaniasis in a disease-endemic area in Bihar, India. Am JTropMed Hyg.2010; 83(3): 502–506. 10.4269/ajtmh.2010.09-0345 PMC2929041 20810810

[49] Hasker E, Malaviya P, Gidwani K, Picado A, Ostyn B, Kansal S. Strong association between serological status and probability of progression to clinical visceral leishmaniasis in prospective cohort studies in India and Nepal. PLoS Negl Trop Dis.2014; 8(1): e2657 10.1371/journal.pntd.0002657 24466361 PMC3900391

[50] Costa SR, D'Oliveira A Júnior, Bacellar O, Carvalho EM. T cell response of asymptomatic Leishmania chagasi infected subjects to recombinant leishmania antigens. Memorias do Instituto Oswaldo Cruz. 1999;94(3): 367–370. 10348984 10.1590/s0074-02761999000300015

